# Chiara Silvestrini

**Published:** 2007-02-12

**Authors:** Mary E. Edgerton

**Affiliations:** M.D. Anderson Cancer Center

I had expected to continue to see Chiara Silvestrini all throughout my life. I had expected that we would continue to compare our experiences as scientists, as women scientists, and as women. Chiara was born in Rome, Italy and lived there all her life. She studied biochemistry and fast reactions. I was born in Austin, Texas, and moved many times. We met while I was a graduate student studying at the University of East Anglia in Norwich, U.K., and she was a visiting scientist. She had a smile with a wink that put you right at ease. She had a lovely Italian accent and would say things like “I am easily seduced by cheesecake.” Or “I hope you will come and visit me in Rome and I will give you a little assay of what life is like there.” When I dried out a separation column in the lab, she smiled and said “It is not the first time a column has ever run dry, nor will it be the last.” That line I have repeated over and over, exchanging the word column with something else to help someone else through a difficult moment.

I visited Rome often. We had a little ritual-the day before I left she would take me to the fountain of Trevi to throw my coin in, insuring my return. At one point I was caught up with life and did not go back every year as I had while I was living in England. Chiara married and had two very lovely sons. I decided to attend medical school and was buried in books. I wrote a letter but did not get an answer, which was not unusual for us. After I finished my residency, I used to the internet to look her up. Email would be easier for us to communicate. I could find papers published up to a certain year, and then no more. I worried about her scientific career-why wasn’t she publishing papers. Out of respect I kept trying to find her email address instead of her husband’s, to locate her. Finally, I gave up and I emailed her husband in Rome. The next day there were two emails from him-I was thrilled. I opened the first in my queue, the second he had sent. It was an “oops” email with the attachment, a wonderful photograph of Chiara smiling-and she had such a beautiful and warm smile. I was so excited. I opened up the next one. I was stunned. She had died a few years before of breast cancer.

It is hard to mourn a friend who is not always close by. It is not a change in the activities of daily living, but there is a big empty feeling where there used to be someone that you always expected to be there. I had the great fortune of visiting Italy shortly after I contacted Chiara’s husband. I met her two wonderful sons, and had a delightful dinner with them and with Alfredo, her husband. She would be proud of them.

I miss her. Now, I want us all to be able to say one day, “It is not the first time I have lost a friend to cancer, but it is the last time. “

